# Simultaneous Spectrophotometric Estimation of Paracetamol and Metoclopramide Hydrochloride in Solid Dosage Form

**DOI:** 10.4103/0250-474X.43014

**Published:** 2008

**Authors:** S. J. Wadher, P. R. Pathankar, Manisha Puranik, R. O. Ganjiwale, P. G. Yeole

**Affiliations:** Department of Pharmaceutical Chemistry, Institute of Pharmaceutical Education and Research, Borgaon (Meghe), Wardha- 442 001, India

**Keywords:** Paracetamol, metoclopramide, simultaneous spectrophotometric, isoabsorptive methods

## Abstract

Two simple, accurate, rapid and economical spectrophotometric methods have been developed for the simultaneous determination of paracetamol and metoclopramide hydrochloride from tablet dosage form. The first method developed employs formation and solving simultaneous equations using 248.6 nm and 275.6 nm as two wavelengths for formation of equations. Second method is absorbance ratio in which wavelengths selected were 265.6 nm as isoabsorptive point and 275.6 nm as λmax of paracetamol. Both the drugs and their mixtures obey Beer-Lamberts law at selected wavelengths at given concentration range. The methods have been validated statistically and by recovery studies. The results of analysis have been validated statistically and by recovery studies.

Paracetamol is chemically N-(4-hydroxyphenyl) acetamide. It is used mainly as antipyretic^1^. Metoclopramide is 4-amino-5-chloro N-(2-diethylaminoethyl)-2-methoxy benzamide. It finds its use as antiemetic^2^. Literature survey reveals that gas chromatography^3^, HPLC^4^, titrimetric^5^ and densitometric^6^ methods are available for the determination of paracetamol and spectrphotometric^7^–^8^ HPLC^9^ and ^1^H NMR spectroscopic^10^ methods for the determination of metoclopramide. The review of literature revealed that no method is yet reported for the simultaneous determination of both the drugs in combined dosage form. This paper describes two simple, rapid, accurate, reproducible and economical methods for the simultaneous determination of metoclopramide and paracetamol from tablet formulation.

A Shimadzu UV/Vis spectrophotometer-2401 with UVPC software and with 10 mm matched quartz cell was used for experiments. All the chemicals and solvents used during project work were of analytical grade. All the solutions were filtered using Whatman filter paper no. 41. Simultaneous equation method was employed as the method I. Pure drug sample of metoclopramide and paracetamol were dissolved separately in methanol so as to give several dilutions of standard in the concentration range 0-30 μg/ml of metoclopramide and paracetamol. All dilutions were scanned in the wavelength range of 400-200 nm. Fig. 1 represents the overlain spectra of both the drugs. Two wavelengths selected for the formation of simultaneous equation are 248.6 nm and 275.6 nm (λ max of paracetamol and metoclopramide) and following equations were used Cx= 179.76 A_2_ -514.12 A_ 1_/445902.57 (1) and Cy= 165 A_1_-925.14 A_2_/445902.57 (2), where, A_1_ and A_2_ are absorbance of sample solution at 248.6 nm and 275.6 nm, respectively, Cx and Cy are concentration (in g/100 ml) of paracetamol and metoclopramide, respectively in sample solution. Molar absorptivities determined for metoclopramide at 248.6 nm and 275.6 nm are 179.96 and 514.12, respectively, and molar absorptivities determined for paracetamol at 248.6 nm and 275.6 nm are 165 and 925.14, respectively.

**Fig. 1:**
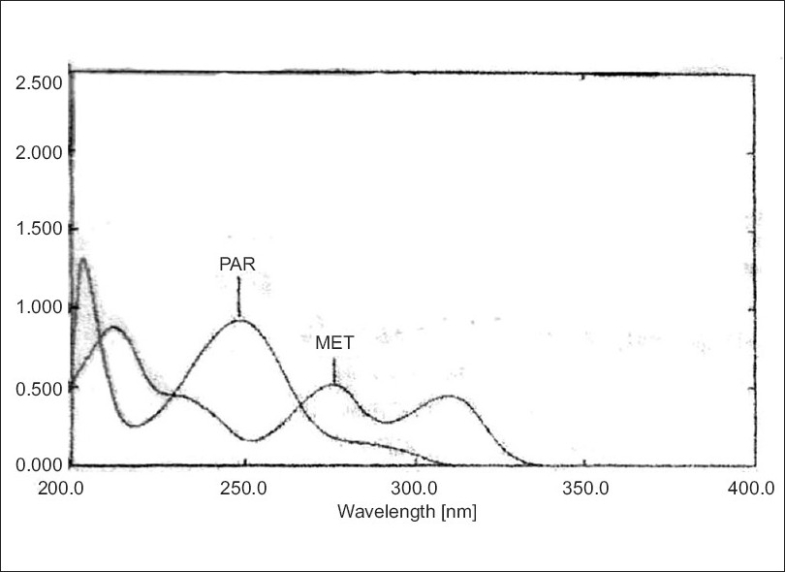
Overlain spectra of paracetamol (PAR) and metoclopramide (MET)

For the estimation of drugs from the commercial formulation, twenty tablets of brand Paramet (Wallace Pharmaceutical, Goa) containing 5 mg of metoclopramide (MET) and 500 mg of paracetamol (PAR). Twenty tablets were weighed accurately and average weight per tablet was calculated. Tablets were ground to a fine powder. A quantity equivalent to 100 mg of PAR was transferred to a 100 ml volumetric flask. MET present in this tablet powder was 1 mg, which could not be found out accurately due to low absorbance; hence to increase the accuracy, accurately weighed 99 mg pure drug sample of MET was transferred to the same volumetric flask. The powder was dissolved in 50 ml methanol with vigorous shaking and volume was made to mark with methanol. The solution was further diluted to get final concentration of 10 μg/ml of PAR (or MET). The absorbance of the solution was measured at 248.6 nm and 275.6 nm and concentration of the two drugs was calculated using Eqns. 1 and 2. The results of tablet formulation are shown in Table 1.

**TABLE 1:** RESULTS OF ANALYSIS OF COMMERCIAL TABLETS

Tablet	Label claim mg/tablet		% Estimated*			
		
	MET	PAR	Method I		Method II	
			
			MET	PAR	MET	PAR
Paramet	5	500	99.45±0.65	100.34±0.84	100.31±1.29	99.51±09

* Mean±SD for five determinations, MET is metoclopramide and PAR is paracetamol

In isoabsorptive point was employed as method II, in which the absorbance was measured at two wavelengths, one being the isoabsorptive point of the two components and other being the wavelength of maximum absorption of one of the two components. From the overlain spectra of two drugs absorbances were measured at selected wavelength i.e. 265.6 nm isoabsorptive point and 275.6 nm, λmax of MET fig. 1.

The absorbance and absorptivity values at the particular wavelengths were calculated and substituted in the following equation; to obtain the concentration Cx= (Qm-Qy)×A/(Qx-Qy)×ax_1_ (3) and Cy= A/ax_1_-Cx (4); where, Cx and Cy are concentration in g/100 ml of PAR and MET, respectively. A is the absorbance of mixture at isoabsorptive point; ax_1_ is the absorbtivity of PAR at isoabsorptive point. Qx was obtained by using the equation, (absorptivity of PAR at 275.6 nm)/ (absorptivity of MET at 265.6 nm); Qy was obtained from (absorptivity of MET at 275.6 nm)/ (absorptivity of PAR at 265.6 nm). Qm from (absorbance of sample at 275.6 nm/absorbance of sample at 265.6 nm)

Tablet solution was prepared in methanol as described earlier and was further diluted to obtain the solution having concentration 10 μg/ml of PAR (or MET). This solution was then analyzed to obtain the spectra and absorbance value at 275.6 nm and 265.6 nm were noted. These values were then equated in above mentioned Eqns. 3 and 4 and the concentration of each drug was calculated and results of tablet formulation are shown in Table 1.

To study the accuracy, reproducibility and precision of the above proposed methods, recovery studies were carried out by addition of standard drug to pre-analyzed sample. Results of recovery studies were found to be satisfactory and reported in Table 2. The value of standard deviation was satisfactorily low and recovery was close to 100% include the reproducibility and accuracy of both the methods.

**TABLE 2:** RECOVERY STUDY DATA

Method	Concentration in sample (μg/ml)		Concentration added (μg/ml)		Amount recovered*		%recovery*	
				
	MET	PAR	MET	PAR	MET	PAR	MET	PAR
I	5	5	5	5	4.90	4.92	98.00	98.40
	10	10	10	10	9.83	10.01	98.30	100.10
	15	15	15	15	15.10	14.97	100.66	99.80
II	20	20	5	5	5.01	4.99	100.20	99.80
	25	25	10	10	10.05	10.10	100.50	101.0
	30	30	15	15	14.80	15.20	98.66	101.33

* Mean of five determinations. MET is metoclopramide and PAR is paracetamol

The first method employing simultaneous equation is a very simple method and can be employed for routine analysis of these two drugs in combined dosage form using simple instrumentation. Once the molar absorptions are determined, very little time will be required for routine analysis, as it would only require determination of absorbance of sample at the two selected wavelengths and few simple calculations.

The second method employed the graphic absorbance ratio at two selected wavelength, once the absorbance is determined. The reported methods were found to be accurate, simple and rapid. Hence it can be employed for routine analysis of both drugs in quality control laboratories.
